# Diagnosis of Insulation Condition of MV Switchgears by Application of Different Partial Discharge Measuring Methods and Sensors

**DOI:** 10.3390/s18030720

**Published:** 2018-02-28

**Authors:** Fernando Álvarez Gómez, Ricardo Albarracín-Sánchez, Fernando Garnacho Vecino, Ricardo Granizo Arrabé

**Affiliations:** Department of Electrical Engineering, Escuela Técnica Superior de Ingeniería y Diseño Industrial (ETSIDI), Universidad Politécnica de Madrid, Ronda de Valencia 3, 28012 Madrid, Spain; ricardo.albarracin@upm.es (R.A.-S.); fernandog@lcoe.etsii.upm.es (F.G.V.); ricardo.granizo@upm.es (R.G.A.)

**Keywords:** partial discharges, insulation diagnosis, switchgears, UHF antennas, HF sensors

## Abstract

Partial discharges (PD) measurement provides valuable information for the condition assessment of the insulation status of high-voltage (HV) electrical installations. During the last three decades, several PD sensors and measuring techniques have been developed to perform accurate diagnostics when PD measurements are carried out on-site and on-line. For utilities, the most attractive characteristics of on-line measurements are that once the sensors are installed in the grid, the electrical service is uninterrupted and that electrical systems are tested in real operating conditions. In medium-voltage (MV) and HV installations, one of the critical points where an insulation defect can occur is inside metal-clad switchgears (including the cable terminals connected to them). Thus, this kind of equipment is increasingly being monitored to carry out proper maintenance based on their condition. This paper presents a study concerning the application of different electromagnetic measuring techniques (compliant with IEC 62478 and IEC 60270 standards), together with the use of suitable sensors, which enable the evaluation of the insulation condition mainly in MV switchgears. The main scope is to give a general overview about appropriate types of electromagnetic measuring methods and sensors to be applied, while considering the level of detail and accuracy in the diagnosis and the particular fail-save requirements of the electrical installations where the switchgears are located.

## 1. Introduction

The presence of PD activity in insulation elements is one of the main causes that can lead to failure in HV electrical systems. PD measurements provide valuable information for assessing the condition of the insulation systems of power cables, power transformers, rotating machines, GIS substations, and metal-clad switchgears [1,2,3,4,5,6], thus contributing to their quality assurance.

Among the PD measuring techniques available (electrical, acoustic, optical, and analysis of chemical by-products) [7,8,9,10,11], the electrical method is the most widely used because of its effectiveness. Despite the usefulness of this technique, when on-line PD tests are carried out, the following drawbacks have to be addressed in order to perform a proper diagnosis: the existence of high levels of background electrical noise, the simultaneous presence of various PD sources, and the difficulty of identifying the defects and of determining their location. To overcome these difficulties, adequate selection of the measuring techniques with the appropriate sensors and the implementation of effective signal processing tools is essential to achieving a correct evaluation of the insulation elements condition [12,13].

Several studies have been conducted with the purpose of achieving accurate diagnosis in on-line PD measurements in HV installations, with most of them being focused on wideband acquisitions through applying distinct methods and sensors [14,15]. With regard to the specific developments related to PD measurements in MV switchgears, an interesting approach to filter on-site electrical noise acquired with non-invasive sensors is presented in [16]. Furthermore, in [17] a sensitivity test is performed for the characterization of a built-in sensor and of a non-invasive one, which are used for PD measurements in switchgears. In [18], an interesting practical development, consisting of a portable PD detection system that implements an ultra-high-frequency (UHF) method, is applied. Although the previous studies reveal important contributions for the improvement in PD measurements performed in MV switchgears, in none of them a characterization of distinct measuring techniques applied to these assets or a comparison among them has been carried out. The research presented in this paper is focused on the analysis of different measuring methods, together with their corresponding sensors, that are commonly applied for the assessment of the insulation condition of MV switchgears. In particular, two non-conventional electrical methods considered in the technical recommendation IEC 62478 [19] and their sensing elements are characterized and evaluated. Furthermore, the so-called conventional electrical method described in IEC 60270 [20] is also considered.

In Section 2, technical details about the measuring methods and sensors studied and implemented in this research are presented. In addition, the technical specifications of the measurement instruments used in the experimental part of this study are described. In the Section 3, the laboratory setup carried out to perform the PD tests for the characterization of the proposed measuring methods is shown. The experimental measurements conducted and the discussion of the results are presented, respectively, in Section 4 and Section 5. Finally, the conclusions of the research are considered in Section 6.

## 2. Measuring Methods, Sensors, and Measurement Devices

### 2.1. Measuring Methods

In the detection of PD pulses by electrical methods, two techniques are mainly distinguished: those that apply the conventional method based on the standard IEC 60270 [20,21], in which PD pulses are measured in a frequency range below 1 MHz, and those that implement non-conventional methods based on the technical specification IEC 62478, in which measurements are performed in the high-frequency (HF) (3–30 MHz), very-high-frequency (VHF) (30–300 MHz), and UHF (300 MHz–3 GHz) ranges [7,19].

The conventional method is used as reference for the quality assurance of HV elements. This method, although suitable for laboratory tests, is inappropriate for on-line measurements, since on site the electrical noise signals are normally very high in the measuring frequencies specified in the IEC 60270 standard (≤1 MHz). In order to avoid such difficulty and achieve an appropriate sensitivity in on-line measurements, non-conventional methods with larger frequency ranges and bandwidths than those specified in the previous standard are needed. Thus, an approach implemented for on-line PD measuring or monitoring in HV systems is based on the acquisition of signals in the HF range by using high-frequency current transformers (HFCTs). These non-invasive sensors are normally placed in the grounding conductors of the earthing network [15,22]. Furthermore, another approach for PD measuring or monitoring is based on the acquisition of signals in the UHF range by using different types of antennas [23]. These antennas can be classified as internal (invasive) or external (non-invasive) sensors, according to whether they are mounted inside or outside the HV equipment. UHF sensors are normally coupled in the resonant metallic chambers of GIS compartments [24], power transformers [2], and metal-enclosed switchgears [18]; in addition they are also coupled in cable accessories [15] and rotating machines [25].

In the research presented in this paper, the two non-conventional approaches introduced previously have been considered for their implementation in on-line PD measuring or monitoring applications for MV switchgears.

The measuring method that operates in HF with HFCT sensors enables autonomous evaluation of the insulation condition, not only of the switchgears to be controlled but also of the installation where these switchgears are in operation. This solution is of interest when a general oversight of the facility is pretended. However, in order to discriminate if a detected defect is within a specific switchgear, further assisted analysis is required. The advantages of applying this method are as follows:

- High sensitivity is obtained when the sensors are located close to the PD source and when they are hundreds of meters from it. Hence, as mentioned before, with this method a high field of vision is achieved and use of a few measuring units positioned in strategic points in a MV installation is sufficient for its supervision.
- If two or more HFCT sensors are placed in a MV or HV installation, the measurement of the PD pulses with a common time reference allows the location of the defects by the time-of-flight analysis.
- When a HFCT sensor is installed in the earth connection of a cable terminal connected to a certain asset (e.g., a power transformer or a MV switchgear), the analysis of the pulses polarity enables it to be discriminated if a PD source is upstream or downstream of that connection [26,27].

The measuring method that operates in UHF implementing antennas as sensors is focused on the autonomous supervision of the insulation condition of the elements integrated in the proper individual switchgears (including the cable terminations connected to them) but not in the rest of the electrical installation where these assets are operating. This solution is of interest for PD detection in switchgears that function in critical MV installations such as, for example, in those that provide electrical energy to major electrical consumers, hospitals, airports, metallurgical industries, etc. The advantages of applying this method are as follows:

- High immunity to electric noise interferences and corona discharges in air, since the frequency spectrum of these signals in the UHF range is very low and in some emplacements negligible.
- High sensitivity in PD detection inside the metal enclosed switchgears due to the inner electrical resonance and low-inherent losses.
- Possibility of assuring that when an insulation defect is detected with an antenna placed in a switchgear, this defect is confined inside this switchgear (including the cable terminations connected to it).

In the research presented in this paper, a measuring method that operates in UHF (measuring with antennas) and another that operates in HF (using HFCT sensors) are proposed for their characterization and evaluation. In addition, the study has been completed with the realization of measurements by applying the conventional method according to IEC 60270 standard. This last method was implemented in order to provide reference measurements for comparative purposes. The main technical aspects of these methods are presented below. A summary of the measuring methods and sensors implemented, and of the frequency ranges used, is shown in Table 1.

Method one. The acquisition is performed in UHF with an antenna whose output is connected directly to a measuring instrument designed to measure in this frequency range. Both the antenna and the measuring instrument are designed to measure PD on-line mainly in MV switchgears. The instrument used with this method consists of an inexpensive radio frequency (RF) tuner that autonomously discriminates electrical noise and PD pulses, analysing the acquired signals in three frequency bands by using the discrete Fourier transform [28]. With this method, when PD activity is detected an alarm is automatically triggered, and the type of defect is autonomously identified. The defect identification is performed by the analysis of the acquired signals in the frequency domain [28]. When a defect is detected in a switchgear, it can be assured that this defect is inside that switchgear; however, it is not possible to discriminate if the defect is on the switchgear side or in a cable termination connected to it. The subsequent evaluation of the measurement results is performed by analysing the appearance of PD activity in a time diagram.

Method two. The acquisition is performed in HF with a HFCT sensor whose output is connected directly to a measuring instrument designed to measure in this frequency range. With the instrument used with this method, an autonomous filtering of the background noise is performed on the basis of the wavelet transform in order to detect only PD pulses. With this method, an alarm signal is automatically triggered when a certain level of PD activity is surpassed. However, when this happens, a further analysis of the measurements is required to determine what kind of PD source is involved and its emplacement. Once an insulation defect is identified (analysing the resulted phase resolved PD-PRPD-pattern) and located inside a switchgear (analysing the arrival time of the PD to different HFCT sensors), the analysis of the pulses’ polarity enables the discrimination of whether the defect is in the switchgear side or in a cable termination connected to it.

Method three. The acquisition is performed according to the standard IEC 60270 by means of a coupling capacitor in series with a measuring impedance (quadrupole). The output of the quadrupole is connected to the measuring instrument. When this method is applied, the tests are carried out in the wide band specified in the standard, and the analysis of the measurements is conducted by evaluating the classical PRPD pattern. As mentioned before, this method is inapplicable for on-line PD measurements because the electrical noise on-site is normally very high. However, the acquisition system through a coupling capacitor, plugged into the cable terminals within switchgear cable compartments, can be used to perform on-line measurements when the signals are processed in the HF range [29,30].

Table 1 shows a summary of the PD measuring methods, sensors, and frequency ranges used.

For the study and characterization of the proposed measuring methods, two characteristic insulation defects placed inside a cabinet that simulates the accessible compartment of a MV switchgear were measured separately by applying simultaneously the methods considered. The technical specifications of the sensors and measurement devices used with the proposed methods are described in next subsections. Furthermore, a description of the technical aspects about the cabinet used is presented.

### 2.2. PD Sensors

**Figure 1:**
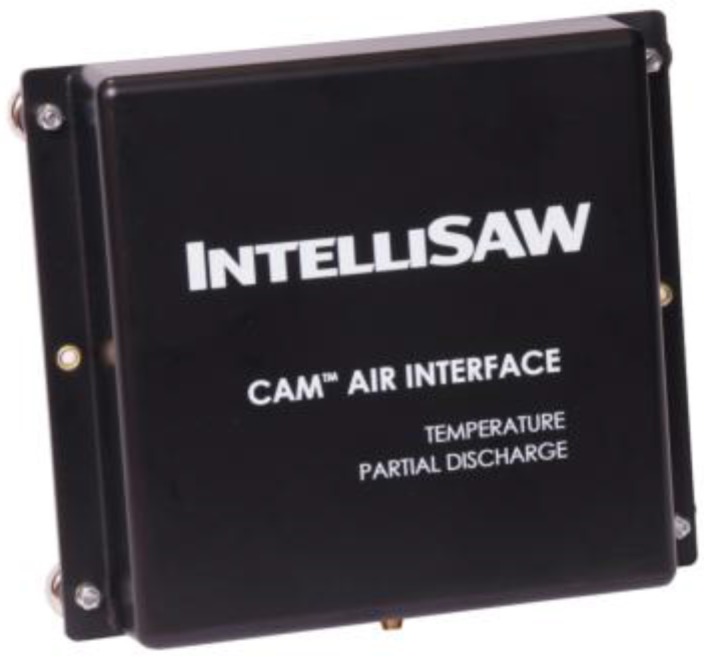
Front view of the UHF antenna.

UHF antenna. This sensor is used to acquire the RF emission radiated from the PD sources in MV switchgears and bus duct chambers. Figure 1 shows a general view of the PD sensor used (IntelliSAW IA-MM-TDP).

The gain of this antenna is +3.5 dBi; it has an impedance of 50 Ω, and its dimensions are 198 × 167 × 43 mm (width, length, and thickness, respectively).

For this antenna, the characterization of the frequency response was determined by measuring the reflection coefficient *S*_11_ that shows how much power is reflected from this sensor. The coefficient *S*_11_ is defined as follows [31]:

(1) $$S_{11}=\frac{Z_{a}-Z_{0}}{Z_{a}+Z_{0}}$$

in which $Z_{a}$ is the load input impedance of the antenna and $Z_{0}$ is the 50 Ω impedance of the acquisition instrument used for its measurement. In this case, this instrument was a vector network analyser (VNA) (Rohde & Schwarz ZNB4).

**Figure 2:**
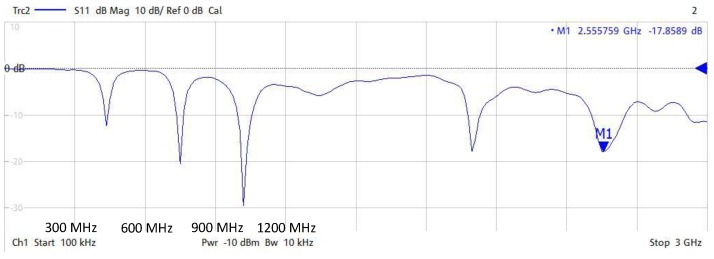
*S*_11_ parameter obtained in free space for the IntelliSAW antenna (300 MHz/div in *x*-axis).

The frequency response of the antenna is shown in Figure 2. According to this, the sensor has a bandwidth span of 300 MHz to 3 GHz. It is noteworthy that this antenna is well matched for *S*_11_ values below −10 dB [32] and that its frequency resonances are mainly located around 425 MHz, 750 MHz, 1100 MHz, 2000 MHz, and 2500 MHz.

**Figure 3:**
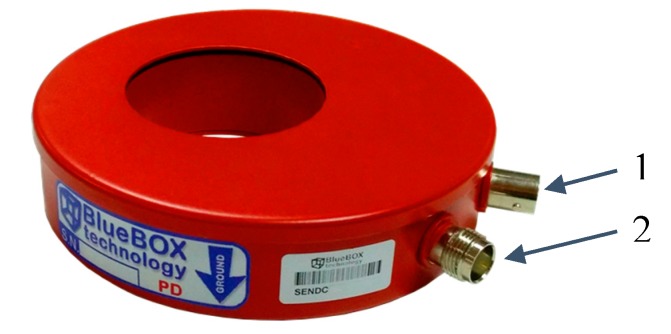
HFCT sensor. (1) BNC output for PD measurement; (2) TNC input for PD checking.

HFCT sensor. This sensor is used to acquire in the HF range the pulses generated in the PD sources of MV and HV equipment [15]. Figure 3 shows a general view of the sensor used (PD-Blue-BOX SEN-DC1) to measure PD pulses in the HF range.

**Figure 4:**
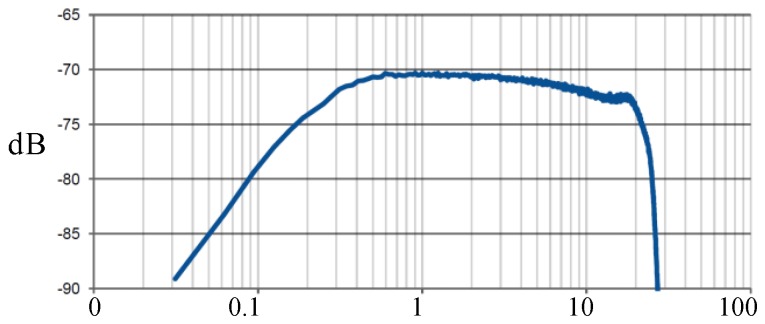
Frequency response of the PD-Blue-BOX SEN-DC1 sensor [15].

Figure 4 shows the frequency response of the sensor used. The bandwidth of this sensor is from 100 kHz to 20 MHz; it has an impedance of 50 Ω, and its dimensions are 110 × 27 mm (external diameter and thickness, respectively).

**Figure 5:**
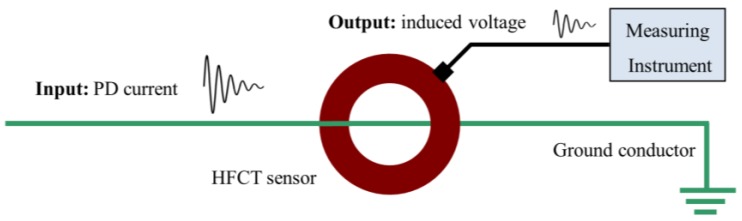
HFCT sensor placed in a ground conductor for PD measurement [15].

This sensor consists of an induction coil with a ferromagnetic core that is suitable for the measurement of transient signals as PD pulses. For this application, the sensor can be modeled as a system in which the input is the current of the PD pulses flowing through it and the output is the induced voltage measured over the input impedance of the measuring instruments (usually 50 Ω), see Figure 5.

The transfer function of these magnetic sensors U = *f*(*B*) can be expressed by Faraday’s law of induction.

(2) $$e=-n\cdot \frac{d\Phi }{dt}=-n\cdot A\cdot \frac{dB}{dt}=-\mu_{0}\cdot n\cdot A\cdot \frac{dH}{dt}$$

In which *Φ* is the magnetic flux passing through the coil of the secondary side, which is formed by a number of turns *n* and presents an area *A*. In the case of a coil with a ferromagnetic core, Equation (2) can be written as follows:

(3) $$e=-\mu_{0}\cdot \mu_{r}\cdot n\cdot A\cdot \frac{dH}{dt}$$

Equation (3) can be expressed as Equation (4), in which the induced voltage in the secondary is proportional to the rate of change of current in the primary, being the mutual inductance *M* between the earth conductor and the secondary, the proportional constant.

(4) $$e=M\cdot \frac{di}{dt}$$

**Figure 6:**
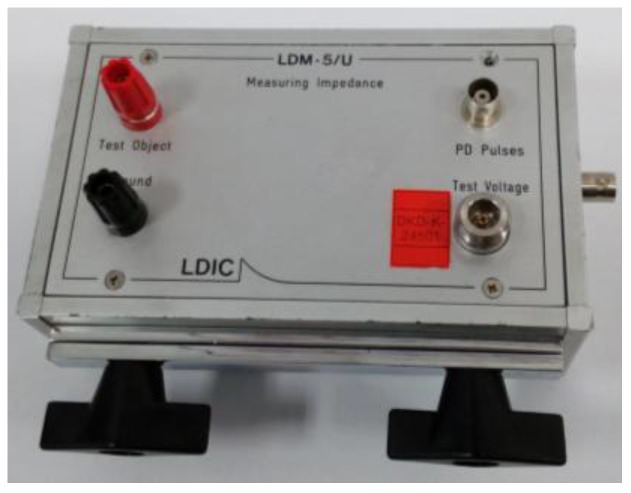
Quadrupole for PD measurement according to the standard IEC 60270.

Measuring quadrupole. This measuring impedance (LDIC LDM-5/U) is used to perform PD tests according to the standard of reference IEC 60270. This sensor has a rated bandwidth from 0 Hz to 10 MHz and an impedance of 50 Ω. Figure 6 shows a general view of this sensor.

### 2.3. PD Measurement Devices and Switchgear Cabinet

PD instrument used with the measuring method one (IntelliSAW IRM-48). This instrument is designed to measure or monitor PD activity, temperature, and humidity in switchgears and in bus duct chambers [28]. Measurement of PD pulses is performed in the UHF frequency range with the antenna described in previous section. The signal processing is made by considering three distinct bands: 225–375 MHz, 475–750 MHz, and 1000–1500 MHz, centred at 300 MHz, 600 MHz, and 1200 MHz, respectively. Although the antenna resonances provided in Figure 2 do not align well with the 300 MHz band in free space, the antenna resonances are often altered in confined spaces, and, experimentally, the 300 MHz band does receive signals in situ. Furthermore, this instrument is integrated with a signal processing tool that enables real-time noise cancellation and the autonomous identification of surface or internal insulation defects. PD activity is quantified by measuring the integrated (cumulative) charge of the pulses over each power cycle. The result calculated for the quantification of PD pulses is mainly used for trending and differential analysis over time. The analysis of the measurements is carried out by evaluating in a time diagram the presence of PD activity and not through the assessment of the PRPD pattern; with this instrument, the acquisition of the reference voltage signal of the HV installation is not necessary.

PD instrument used with the measuring method two (DIAEL-BlueBOX MS Plus). This instrument is designed to perform on-line PD measuring or monitoring, in the HF range in MV and HV installations. This device can also be used to process PD measurements performed in the UHF range by using an UHF-HF frequency converter [15]. The technical characteristics of this instrument are 50 MHz of bandwidth, 100 MS/s of sampling frequency, and 14 bits of vertical resolution. This device is equipped with three processing tools: a noise filter based on the wavelet transform, a defect location software to identify where the PD source is placed, and a PD pulses clustering software that enable identification of different PD sources that are active simultaneously [15]. The analysis of the measurements is carried out by evaluating the resulted PRPD pattern, the arrival time of the PD to different HFCT sensors, and the pulses polarity.

PD instrument used with the measuring method three (Omicron MPD 600). Although this instrument is designed to perform on-line measurements in selectable frequency bands of the HF range, in this study it has been used for PD measurement by applying the conventional method according to the standard IEC 60270. The tests with this device were carried out with a central frequency of 200 kHz and a bandwidth of ±300 kHz.

MV Switchgear cabinet. The experimental measurements presented in Section 4 were developed by considering insulation defects inside a cabinet that simulates the accessible compartment of a MV switchgear, with dimensions of 58.42 × 45.72 × 88.9 cm. The cabinet has the electromagnetic behaviour of a rectangular resonant cavity [33]. Thus, when there is PD activity inside, resonance frequencies are created due to the multiple reflections of stationary waves reflecting on its conductive metal walls [31]. These resonant frequency modes can be calculated ideally as follows [34,35]:

(5) $${\left(f_{rc}\right)}_{mnp}=\frac{1}{2\pi \sqrt{\mu \epsilon }}\sqrt{{\left(\frac{m\pi }{a}\right)}^{2}+{\left(\frac{n\pi }{b}\right)}^{2}+{\left(\frac{p\pi }{c}\right)}^{2}}$$

in which *m*, *n*, and *p* are the sub-indices used to designate the distribution of a stationary wave inside a resonant cavity whose axis are *x*, *y*, and *z*, respectively; *f_rc_* is the resonance frequency of the cavity, $\mu =4\cdot 10^{-7}\;\mathrm{Hm}/A$ the permeability of vacuum, $\epsilon =8.85\cdot 10^{-12}\;F/m$ the permittivity of vacuum, and *a*, *b*, and *c* the dimensions of the cabinet in meters. Assuming that the maximum length of a cabinet corresponds to the *z*-axis propagation, width with the *x*-axis, and height with the *y*-axis, and according to Equation (5), the frequencies in which the cabinet resonates are as follows: 307 MHz, 369 MHz, 416 MHz, 423 MHz, 449 MHz, 470 MHz, 535 MHz, 540 MHz, 609 MHz, 632 MHz, 677 MHz, 704 MHz, 724 MHz, 849 MHz, 1 GHz, 1.2 GHz, and 1.35 GHz. It is noteworthy that below 300 MHz, stationary waves are not propagated inside the cabinet. The ideal frequencies calculated only for the enclosure allow an acceptable approach to the real frequency resonances in the rectangular cavity [31]. It should be noted that when these frequencies are calculated for the enclosure of a real switchgear, the results can slightly shift due to the content inside it, such as bus bars, cables, surge arrestors, and mechanical assemblies that break up the geometry into smaller subsections. Once the resonance frequencies of the cabinet used in the tests have been characterized, it can be checked that the antenna used in the measurements is well adapted to these frequencies. In order to optimize the sensitivity when the measurements are performed in MV switchgears in the UHF range, it should be checked that the antenna used is well adapted to the resonance frequencies of the enclosure where they are installed.

## 3. Experimental Setup

**Figure 7:**
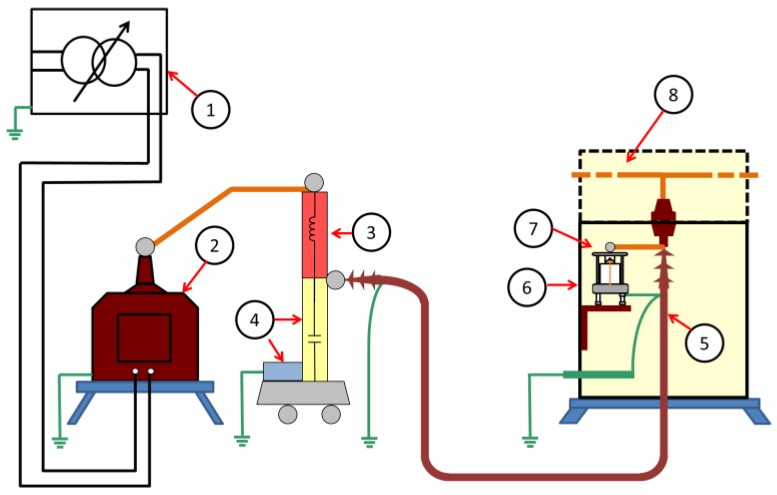
Layout of the experimental setup implemented in the HV laboratory.

For the characterization of the proposed methods to measure PD activity inside MV switchgears, two laboratory tests were performed in measuring real insulation defects. To carry out these tests, an experimental setup was implemented in the HV laboratory of the Universidad Politécnica de Madrid (LAT-UPM). In Table 2, the main elements that make up this setup are provided. Furthermore, these parts are shown in the layout of Figure 7.

With the autotransformer (1), the voltage level generated by the HV transformer (2) is regulated. The noise blocking impedance (3) is a filter that enables the rejection of electromagnetic noise and pulsating interferences coming from the side of the HV transformer; moreover, it prevents a short cut for the PD signals generated through the HV transformer. This blocking impedance, the capacitive divider, and the measuring quadrupole (4) are used to perform PD measurements according to the standard IEC 60270 and to acquire the reference voltage signal for the PD tests. A MV power cable (5) is connected to the MV cabinet (6). Inside the accessible part of the cabinet, test cells with insulation defects (7) are connected in parallel to the MV cable termination in order to simulate defects on it. The upper part of the cabinet (8) represents the circuit breaker and bus duct compartment.

## 4. Experimental Measurements

**Figure 8:**
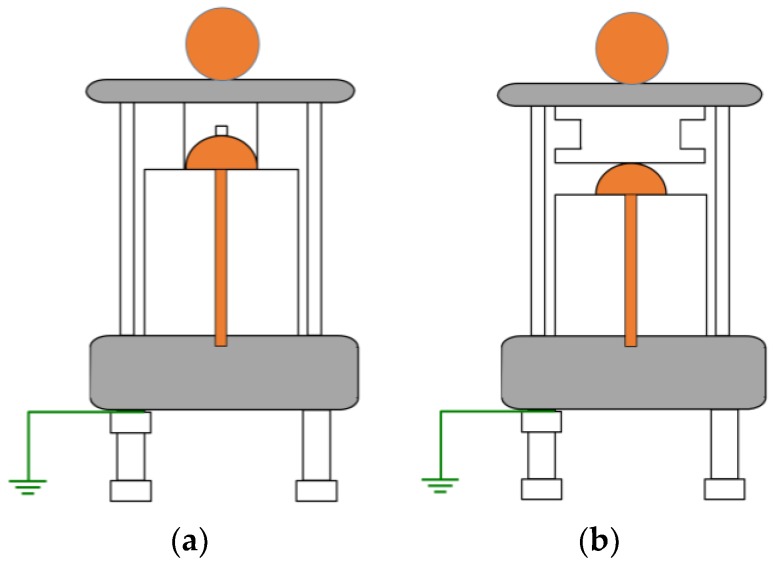
Layout of the test cells. (**a**) Internal defect and (**b**) surface defect.

With the aim of characterizing the capabilities of the measuring methods and sensors proposed in Section 2, two characteristic insulation defects were measured at different voltage levels. The first one was an internal defect in a solid dielectric and the second was a surface defect in air. These defects were integrated in the test cells shown in Figure 8 and represent characteristic insulation defects that can be found on-site in MV switchgears.

**Figure 9:**
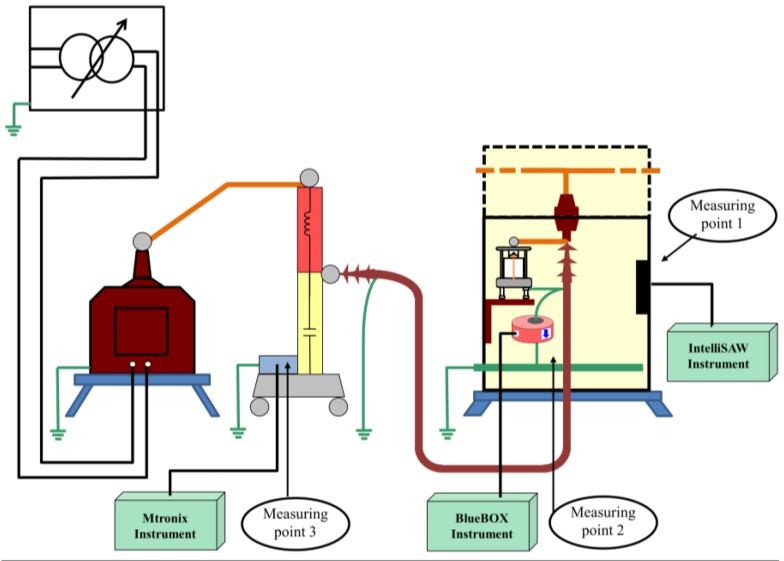
Measuring points, sensors, and instruments indicated in the experimental setup.

The measurements were performed by applying the proposed methods simultaneously for each test cell in the laboratory setup described in Section 4. In Figure 9, the same setup is presented but in this case the measuring points where the sensors were installed and the instruments connected to them are indicated. For the measurements, the test cells were installed consecutively inside the cabinet. As mentioned before, these cells are connected in parallel with the cable termination with the aim of simulating an insulation defect on it.

For the measuring method one in UHF, the antenna was positioned on the right side of the cabinet (measuring point one); this antenna was connected directly to the UHF PD instrument. The acquisitions in HF corresponding to method two were performed with the HFCT sensor installed in the grounding conductor of the cable termination (measuring point two). The output of this sensor was connected to the HF PD instrument. Lastly, for the measuring method three, the acquisition was carried out according to the standard IEC 60270 by means of a coupling capacitor and a quadrupole (measuring point three). The output of this quadrupole was connected to the corresponding PD instrument. By way of a summary, Table 3 shows, for each applied method, the sensor implemented, its emplacement, and the measuring frequency range used. The results of the measurements performed for each insulation defect are presented in the subsections that follow.

### 4.1. Measurement of the Internal Defect

**Figure 10:**
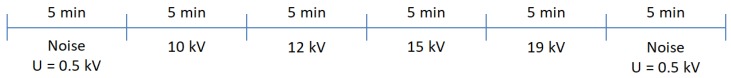
Time intervals and applied voltages for the measurement of the internal insulation defect.

The internal defect was measured at different voltage levels for 30 min. Over the first five minutes and the last five minutes of the test, as the voltage applied was 0.5 kV, in these intervals only the background noise was measured. The inception voltage of the discharges was 10 kV, and the voltage levels for the measurement of the defect were 10 kV, 12 kV, 15 kV, and 19 kV. At each of these steps the voltage was maintained for five minutes. The time intervals and the corresponding applied voltages are shown in Figure 10. It was observed that the higher the applied voltage level is, the higher the pulse activity is both in amplitude and in appearance rate (pulses per cycle).

**Figure 11:**
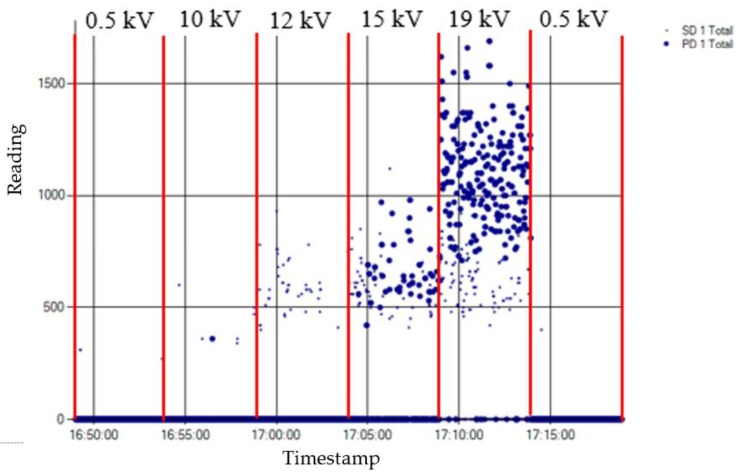
Result of the measurement of the internal defect applying method one.

The result obtained for the measurement performed by applying method one is shown in Figure 11. The analysis of this measurement is carried out by the evaluation of the PD activity appearance in a time diagram, which enables the detection and identification of the insulation defect. Consequently, this method directly offers time series with the result of the measurement.

**Figure 12:**
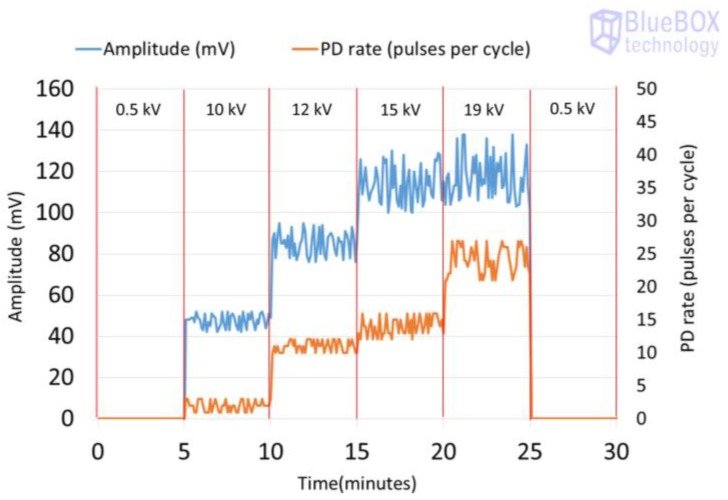
Time series corresponding to the PD activity (amplitude and rate) measured for the internal insulation defect when method two is applied.

The results obtained for the measurements performed by applying methods two and three are shown in Table 4. The analysis of these measurements is carried out by the evaluation of the classical PRPD patterns that enable the detection and identification of the insulation defects. The values of amplitude or charge and PD rate obtained can be visualized to analyse the time series. In Figure 12, the PD time series corresponding to the measurement performed with method two is shown.

As the study is focused on on-line PD measurements in MV switchgears, the results obtained with method three are not studied in detail in the following subsections. These reference measurements were performed in the laboratory for the quantification of the PD activity and for comparative purposes concerning PD detection and PD rate.

• *Evaluation of the Results for the Internal Defect*

The evaluation of the results, when methods one and two are applied, is carried out by taking into account the following important criteria considered by electrical utilities and maintenance companies, when a proper supervision of the insulation condition in MV switchgears is sought. These criteria are as follows:

- Regarding the insulation defect detection:
  ◾ Sensitivity in the detection.
  ◾ Analytical capability for the assisted or autonomous detection.
- Regarding the type of defect identification:
  ◾ Analytical capability for the assisted or autonomous defect recognition.
- Regarding the defect location (determination of the MV switchgear where the PD source is present and identification of the defective element inside it):
  ◾ Analytical capability for the assisted or autonomous identification of the affected MV switchgear and for the identification of the defective element (cable termination or rest of components).

Method one. With this method, the PD pulses are autonomously discriminated from the electrical background noise through an analysis of the acquired signals in three frequency bands. The variable background noise (radio frequency interferences (RFI)) is characterized by using the discrete Fourier Transform (DFT) and is intentionally desensitized so that only PD activity above this noise floor is reported. Once the PD activity is automatically detected, an alarm is triggered and the diagnosis software that is used autonomously identifies the type of the measured PD when it is generated in an internal or in a surface insulation defect. In such a way, plant operators can be notified of the type of defect without the requirement of further analysis of the measurements. The identification is performed by the analysis of the frequency spectrum of the acquired signals [28]. When internal discharges are detected, the PD activity is represented in the time diagram with large dots, and when surface discharges are detected the activity is shown with small dots. By analysing the distribution of the PD activity shown in Figure 11, it can be asserted that this method enables the detection of PD activity in the voltage level of 10 kV (inception voltage), although this detection was not very significant. With this method, the objective is to perform a trend analysis of the PD activity, so in some cases this solution tends to classify discharges near inception as noise, since they do not present a reproducible discharge repetition [28]. This is done with the aim of avoiding false alarms that may confuse power plant operators; thus, no consistent report of internal PD at the inception voltage should be expected. At 12 kV, although the PD activity is clearly detected, the pulses are not yet identified as internal discharges. The insulation defect was clearly detected and autonomously identified when higher voltage levels (15 kV and 19 kV) were applied. With this method, as the measurement is performed in UHF, it is possible to assure that if an insulation defect is detected with an antenna placed in a switchgear, this defect is confined inside that switchgear (including the cable terminations connected to it). Therefore, this technique allows also the autonomous location in the supervised installation of the MV switchgear where an insulation defect is present.

**Figure 13:**
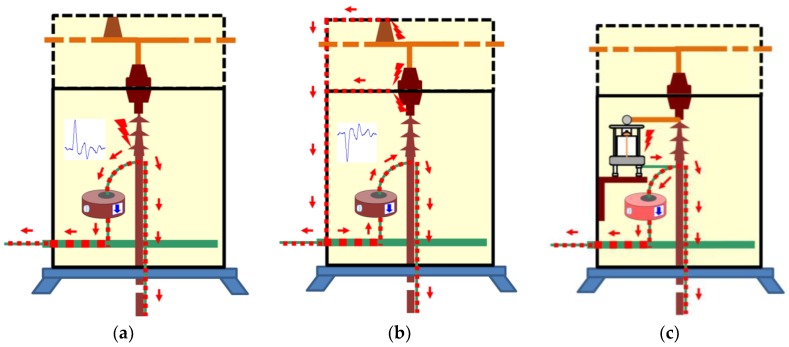
Discrimination of the affected element (cable termination or rest of components). (**a**) Insulation defect in the cable termination. (**b**) Insulation defects in the switchgear elements. (**c**) Measured internal insulation defect simulated in the cable termination.

Method two. With this method, the PD pulses are also autonomously discriminated from the variable electrical background noise by applying a filter based on the wavelet transform. Once the PD activity is automatically detected, an alarm is triggered, and through the diagnosis of the resulted PRPD pattern the type of insulation defect is identified. In this case, by analysing the results shown in the second column of Table 4 and in Figure 12, it can be asserted that this method enables detection of PD activity in the voltage level of 10 kV (inception voltage). Representative patterns of an internal defect were obtained for all the voltage levels applied: PD activity occurs close to the zero crossings and in the increasing intervals of the test voltage; furthermore, the individual PD amplitude values in both half-periods are highly variable. With this method, the location of the switchgear where the insulation defect is present, including the cable terminations connected to it, is performed autonomously by the diagnosis software, through time-of-flight analysis of the pulses measured with two HFCT sensors placed in strategic points of the installation to be supervised [15]. In addition, the analysis software of the technology applied with this method enables it to be determined if the insulation defect (present in the located switchgear) is in a cable termination connected to it or in one of the switchgear elements. The discrimination of the affected element (cable termination or rest of components of the switchgear) is performed, in an assisted way, by the polarity analysis of the pulses measured with the HFCT sensor placed inside the switchgear. For the arrangement of the sensor established in the switchgear (see Figure 9 and Figure 13a), when the defect is in a cable termination, the PD pulses recorded in a positive half-period of the mains reference voltage signal also have a positive polarity. However, when the defect is in a component of the switchgear itself (see Figure 13b), the pulses recorded in this positive half-period have a negative polarity. The pulses polarity changes due to their different travelling path from the PD source to the HFCT sensor. In Figure 13a–c the trajectory of the pulses is shown with a red dashed line. The main path of the PD pulses measured with HFCT sensors placed in MV switchgears is through the shields of the power cables connected to them. For the internal insulation defect measured (see Figure 13c), the pulses polarity in the positive half-cycle is positive, which indicates that the defect is in the cable termination. It is remembered that the test cell was connected in parallel with the cable termination with the aim of simulating an insulation defect on it.

Method three. The patterns obtained by applying the conventional method according to the standard IEC 60270 are presented in the third column of Table 4. As the measurement was performed in a laboratory with a low level of electrical background noise, with this method the detection of PD activity was possible from the inception voltage (10 kV). In this case, clear and representative patterns of an internal defect were obtained for all the voltages applied: PD occur close to the zero crossings (with 10 kV and 12 kV), in the zero crossings (with 15 kV and 19 kV), and in the increasing intervals of the test voltages; moreover, the individual amplitude values in both half-periods are highly variable, and there is a certain symmetry when comparing the patterns shape of both half-periods in the logarithmic scale. In this case, for the test voltage of 10 kV, the reference weighted charge value for the corresponding pattern was 336.4 pC. The highest charge values in the positive and negative half cycles were 717 pC and 510 pC, respectively (see Table 4). All the reference values obtained in the complete test performed (PD charge and appearance rate) can be compared with the readings obtained with method one and with the values of amplitude and rate obtained with method two.

### 4.2. Measurement of the Surface Defect

**Figure 14:**
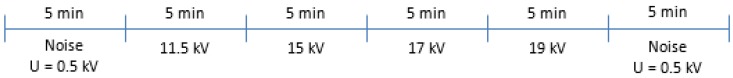
Time intervals and applied voltages for the measurement of the surface insulation defect.

The surface defect was measured at different voltage levels for 30 min. Again, over the first five minutes and the last five only the background noise was measured as the applied voltage was 0.5 kV. The inception voltage of the discharges was 11.5 kV and the voltage levels for the measurement of the defect were 11.5 kV, 15 kV, 17 kV, and 19 kV. The time intervals and the corresponding applied voltages are shown in Figure 14. For this defect too, the higher the applied voltage level is, the higher the pulse activity is, both in amplitude and in rate.

**Figure 15:**
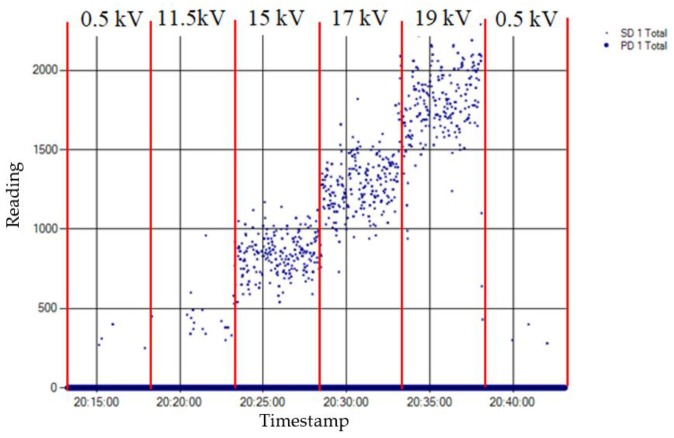
Result of the measurement of the surface defect applying method one.

**Figure 16:**
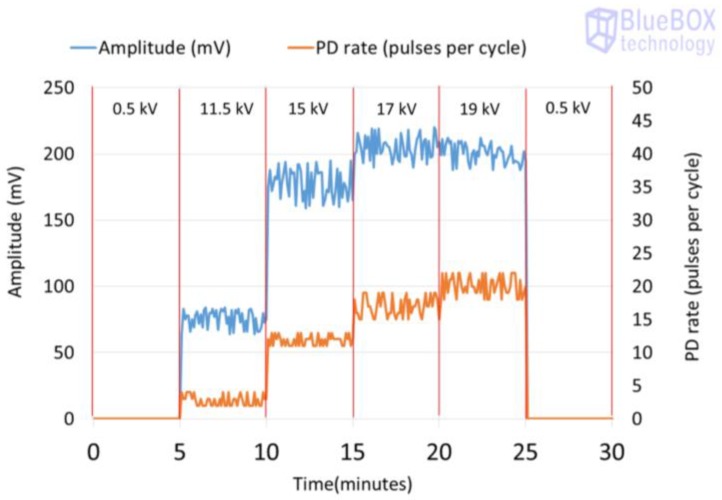
Time series corresponding to the PD activity (amplitude and rate) measured for the surface insulation defect when method two is applied.

The result obtained for the measurement performed by applying method one is shown in Figure 15, and the results for the measurements made by applying methods two and three are shown in Table 5. Figure 16 shows the time series that corresponds to the measurement performed with method two.

• *Evaluation of the Results for the Surface Defect*

The evaluation of the results, when methods one and two are applied, is carried out by taking into account the same criteria that were considered when the internal defect was analyzed.

Method one. By analysing the distribution of the PD activity shown in Figure 15, it can be asserted that this method enables detection of some PD activity in the voltage level of 11.5 kV (inception voltage). However, the results obtained appear to be more reliable when the PD repetition rate is higher; so, also with the surface defect, the method tends to reject PD signatures with small repetition rates near the inception voltage. This is an expected consequence of the DFT analysis performed and the application-specific goal of only reporting well substantiated and actionable PD conditions. Furthermore, as the PD activity is depicted in all voltage levels with small dots, the surface defect was successfully identified.

Method two. By analysing the results shown in the second column of Table 5 and in Figure 16, it can be asserted that this method enables the detection of PD activity in the voltage level of 11.5 kV (inception voltage). Furthermore, representative patterns of a surface defect can be visualized for all the voltages levels applied: PD activity occurs in the increasing intervals and also in the peak of the test voltage, and the individual PD amplitude values in both half-periods (especially in the positive one) are variable; furthermore, there is an asymmetry when comparing the patterns shape of both half-periods. For this insulation defect, the defected element was also successfully identified, as the polarity of the measured pulses in the positive half-cycle was positive, and the defect was simulated in the cable termination.

Method three. The patterns obtained by applying the conventional method according to the standard IEC 60270 are presented in the third column of Table 5. The detection of PD activity was also possible from the voltage level of 11.5 kV. Again, clear and representative patterns of a surface defect are shown for all the voltages applied. All the reference values measured with this method (PD charge and rate) can be compared with the readings acquired with method one and with the values of amplitude of method two.

## 5. Discussion

In this section, the results obtained for the two methods proposed for carrying out on-line PD measurements for the supervision of MV switchgears are discussed. In order to provide a general overview of their capability to monitor the insulation condition of these assets, the evaluation criteria presented in previous section are reviewed in Table 6. The criteria considered for the evaluation are examined when the two methods are applied separately and jointly.

When method one was applied, the insulation defects were autonomously detected. Although with this method the sensitivity in the detection was slightly lower, the autonomous identification of the type of defect was successfully performed, especially when the PD rate was significant. When method two was applied, the insulation defects were also autonomously detected; however, for their identification an assisted analysis was required. Once an insulation defect is detected, with both methods the automatic location of the affected switchgear in the supervised installation is possible. An analytical capability of method two enables it to be determined, in an assisted way, if the insulation defect located in a certain switchgear is in the cable terminal connected to it or in one of the rest of components.

Through analysing the results obtained for each technology according to the considered technical criteria, it can be ascertained that both have been proven to be suitable for the supervision of the insulation status of MV switchgears. The study performed enables the assessment of the main features of both methods that are considered when a proper supervision of MV switchgears is required. As each technique stands out in different capabilities, it can be stated that both are complementary. Thus, an important aspect to be considered is that with the simultaneous application of both technologies an enhancement in the autonomous supervision of MV switchgears can be achieved. In the last row of Table 6, it can be verified that when both methods are applied together the overall results improve importantly.

## 6. Conclusions

This research is focused on the study and characterization of different PD measuring techniques and sensors, which are applied in on-line measurements for the evaluation of the insulation condition of MV switchgears. Non-conventional methods, with wider frequency ranges and bandwidths than those specified in the standard IEC 60270, are normally applied in on-site PD measurements to avoid mainly the inconvenience of the presence of electrical noise.

An approach that is implemented in on-line PD measuring or monitoring applications of MV switchgears is based on the acquisition of the signals in the UHF range using an antenna as a sensor. Another approach is based on the acquisition of the signals in the HF range using a HFCT sensor. Two measuring methods based on such approaches have been studied and characterized in this research.

In some applications, particularly those related to high-value and system-critical assets, the cost of analysis is readily offset by the improved reliability. Such applications have relied upon human analysis of large amounts of data to determine the asset health of mainly power transmission networks. While these approaches are suitable for large and extremely critical assets, they are not scalable to the millions of distribution assets and the balance of plant assets worldwide. Thus, the measuring methods considered in this paper are based on practical solutions for the autonomous supervision of distribution switchgears operating in on-site distribution installations.

By considering the technical features and the capabilities of the proposed technologies, evaluated and discussed in previous section, the following conclusions are drawn regarding on line-PD measurements in MV switchgears.

The study of the capabilities considered for the evaluation of both methods offers an overview of their main characteristics, which is useful when developing an application for PD monitoring in MV switchgears.

Although each method stands out as regards different aspects, it can be stated that both are efficient for the supervision of the insulation condition of MV switchgears.

In addition, an important aspect to be highlighted, after the completion of this research, is that through the joint application of both methods, electrical utilities and maintenance companies have the capacity to increase significantly the efficiency in the autonomous supervision of electric power distribution facilities.

## Figures and Tables

**Table 1:** Measuring methods and sensors implemented and frequency ranges used.

Measuring Method	Measuring Technique	Sensor	Frequency Range
1	UHF	UHF Antenna	UHF
2	HF	HFCT	HF
3	IEC 60270	Quadrupole	<1 MHz

**Table 2:** Main components of the experimental setup.

Number of Element	Component of the Setup
1	Autotransformer (voltage regulator), 0–250 V, 3 kVA
2	HV transformer 110 V/66 kV, 600 VA
3	Blocking impedance
4	Capacitive divider and measuring quadrupole (according to IEC 60270)
5	MV power cable
6	Accessible part of the MV metal cabinet
7	Test cell with insulation defect
8	Circuit breaker and bus duct compartment

**Table 3:** Applied methods, sensors, measuring points, and measuring frequency ranges.

Measuring Method	Sensor	Measuring Point	Measuring Frequency Range
UHF	UHF Antenna	1	225–375, 475–750 and 1000–1500 MHz
HF	HFCT	2	10 kHz–20 MHz
IEC 60270	Quadrupole	3	<1 MHz

**Table 4:** Results of the measurement of the internal defect applying methods two and three.

Voltage Level	Method Two (HF) Sensor: HFCT Equipment: BlueBOX		Method Three (IEC 60270) Sensor: Quadrupole Equipment: Mtronix	
10 kV	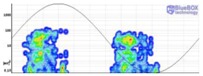	Rate: 2 PDs/cycle Amplitude: 47 mV Max. amplitude by semi period: “+”: 105 mV “−”: 60 mV	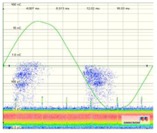	Rate: 3 PDs/cycle Charge: 336 pC Max. charge by semi period: “+”: 717 pC “−”: 510 pC
12 kV	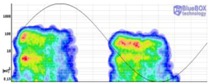	Rate: 11 PDs/cycle Amplitude: 85 mV Max. amplitude by semi period: “+”: 140 mV “−”: 85 mV	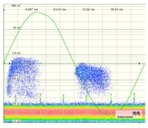	Rate: 9 PDs/cycle Charge: 527 pC Max. charge by semi period: “+”: 800 pC “−”: 530 pC
15 kV	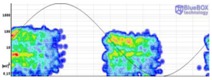	Rate: 14 PDs/cycle Amplitude: 114 mV Max. amplitude by semi period: “+”: 162 mV “−”: 93 mV	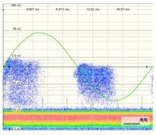	Rate: 12 PDs/cycle Charge: 635 pC Max. charge by semi period: “+”: 970 pC “−”: 720 pC
19 kV	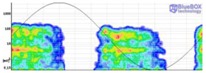	Rate: 24 PDs/cycle Amplitude: 121 mV Max. amplitude by semi period: “+”: 165 mV “−”: 116 mV	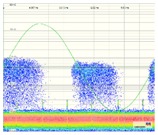	Rate: 20 PDs/cycle Charge: 698 pC Max. charge by semi period: “+”: 1.2 nC “−”: 868 pC

**Table 5:** Result of the measurement of the surface defect applying methods two and three.

Voltage Level	Method Two (HF) Sensor: HFCT Equipment: BlueBOX		Method Three (IEC 60270) Sensor: Quadrupole Equipment: Mtronix	
11.5 kV	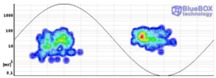	Rate: 3 PDs/cycle Amplitude: 74 mV Max. amplitude by semi period: “+”:101 mV “−”: 112 mV	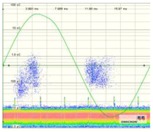	Rate: 3 PDs/cycle Charge: 512 pC Max. charge by semi period: “+”: 1.4 nC “−”: 1.1 nC
15 kV	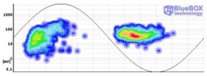	Rate: 12 PDs/cycle Amplitude: 174 mV Max. amplitude by semi period: “+”: 307 mV “−”: 170 mV	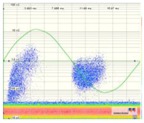	Rate: 10 PDs/cycle Charge: 1.6 nC Max. charge by semi period: “+”: 4 nC “−”: 1.5 nC
17 kV	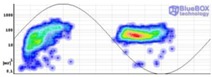	Rate: 17 PDs/cycle Amplitude: 206 mV Max. amplitude by semi period: “+”: 285 mV “−”: 214 mV	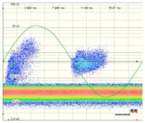	Rate: 15 PDs/cycle Charge: 2 nC Max. charge by semi period: “+”: 4 nC “−”: 1.5 nC
19 kV	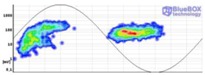	Rate: 20 PDs/cycle Amplitude: 201 mV Max. amplitude by semi period: “+”: 325 mV “−”: 265 mV	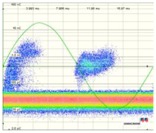	Rate: 17 PDs/cycle Charge: 2.4 nC Max. charge by semi period: “+”: 4 nC “−”: 2.9 nC

**Table 6:** General overview of the proposed methods capabilities.

Method	Insulation Defect Detection		Insulation Defect Identification		Insulation Defect Location (1)		Defected Element Identification (2)	
	Autonomous	Detection (Sensitivity)	Autonomous	Identification	Autonomous	Location	Autonomous	Identification
1 (UHF)	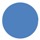	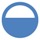	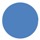	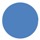	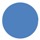	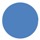	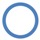	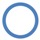
2 (HF)	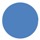	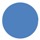	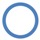	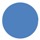	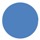	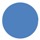	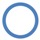	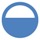
1&2 (UHF&HF)	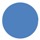	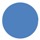	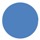	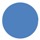	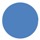	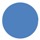	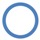	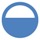

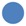
 Achieved 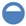 Not totally achieved 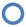 Not achieved. (1) Localization in the supervised installation of the MV switchgear where the insulation defect is present. (2) Identification of the defective element inside the switchgear (cable termination or rest of components).
